# Non-Invasive Evaluation of Cerebral Hemodynamic Changes After Surgery in Adult Patients With Moyamoya Using 2D Phase-Contrast and Intravoxel Incoherent Motion MRI

**DOI:** 10.3389/fsurg.2022.773767

**Published:** 2022-03-22

**Authors:** Feng Gao, Wei Zhao, Yu Zheng, Shihong Li, Yu Duan, Zhenfang Zhu, Ming Ji, Jun Liu, Guangwu Lin

**Affiliations:** ^1^Department of Radiology, Huadong Hospital, Fudan University, Shanghai, China; ^2^Department of Radiology, Second Xiangya Hospital, Central South University, Changsha, China; ^3^Department of Radiology, Chengdu Second People's Hospital, Chengdu, China; ^4^Department of Neurosurgery, Huadong Hospital, Fudan University, Shanghai, China

**Keywords:** moyamoya disease, intravoxel incoherent motion, phase-contrast MRI, Suzuki stage, surgery

## Abstract

**Objective:**

To explore the feasibility of 2D phase-contrast MRI (PC-MRI) and intravoxel incoherent motion (IVIM) MRI to assess cerebrovascular hemodynamic changes after surgery in adult patients with moyamoya disease (MMD).

**Methods:**

In total, 33 patients with MMD who underwent 2D PC-MRI and IVIM examinations before and after surgery were enrolled. Postsurgical changes in peak and average velocities, average flow, forward volume, and the area of superficial temporal (STA), internal carotid (ICA), external carotid (ECA), and vertebral (VA) arteries were evaluated. The microvascular perfusion status was compared between the hemorrhage and non-hemorrhage groups.

**Results:**

The peak velocity, average flow, forward volume, area of both the ipsilateral STA and ECA, and average velocity of the ipsilateral STA were increased (*p* < 0.05). The average flow and forward volume of both the ipsilateral ICA and VA and the area of the ipsilateral VA were increased (*p* < 0.05). The peak velocity, average velocity, average flow and forward volume of the contralateral STA, and the area of the contralateral ICA and ECA were also increased (*p* < 0.05), whereas the area of the contralateral VA was decreased (*p* < 0.05). The *rf* value of the ipsilateral anterior cerebral artery (ACA) supply area was increased (*p* < 0.05) and more obvious in the non-hemorrhage group (*p* < 0.05).

**Conclusion:**

Two-dimensional PC-MRI and IVIM may have the potential to non-invasively evaluate cerebrovascular hemodynamic changes after surgery in patients with MMD. An improvement in the microvascular perfusion status is more obvious in patients with ischemic MMD than in patients with hemorrhagic MMD.

## Introduction

Moyamoya disease (MMD) is a cerebrovascular disease of an unknown etiology characterized by bilateral steno-occlusive changes at the terminal part of the internal carotid artery (ICA) and an abnormal vascular network at the base of the brain (1). Cerebrovascular reconstruction is highly recommended to improve the cerebrovascular hemodynamic status (2). After surgical revascularization, it is very important to evaluate cerebral hemodynamic changes to assess the patency of the bypass and potential complications, such as ischemia and hyperperfusion syndrome (3).

Transcranial Doppler (TCD) has been widely used to evaluate cerebral hemodynamic changes after revascularization surgery. However, it has several inherent disadvantages. TCD can only indirectly evaluate cerebral collateral circulation by monitoring the blood flow of the superficial temporal artery (STA). Moreover, TCD cannot simultaneously evaluate cerebral microvascular perfusion, which is crucial for monitoring the perfusion status after surgery (3). In contrast, phase-contrast MRI (PC-MRI) can directly measure the hemodynamic status of different vessels and has been widely used to evaluate the hemodynamic status of the carotid artery and vertebral artery (VA), obtaining accurate and reliable results (4).

In addition to hemodynamic changes, the perfusion status is another vital factor to monitor the treatment effect. Perfusion imaging modalities, such as CT perfusion imaging and dynamic susceptibility contrast-perfusion weighted imaging (DSC-PWI), are conventional strategies to evaluate cerebrovascular changes. Nonetheless, they require the management of a contrast agent. In this context, MRI perfusion methods without the need for contrast agents, such as blood oxygenation level dependence (BOLD), arterial spin labeling (ASL), and intravoxel incoherent motion (IVIM), are widely investigated as alternative modalities. BOLD is related to tissue oxygenation, and the BOLD MRI signal is a complex function of CBF and is related to basal EtCO_2_ (5, 6). However, BOLD is rarely used to evaluate the perfusion status due to its complexity. Regarding ASL, despite the advantage of non-invasiveness, it is sensitive to arterial arrival delays (7). Conversely, IVIM imaging, a new perfusion evaluation modality, is designed to determine microvascular perfusion and microstructural integrity simultaneously (8) and may be better for detecting the changes in microvascular perfusion and microstructural integrity in patients with MMD after surgery. It has been proven to provide essential information on microperfusion in the tissue; therefore, it is a promising tool for applications in neurological and neurovascular diseases (9, 10).

The ability of PC-MRI to assess cerebral blood flow in patients with MMD, especially to measure the hemodynamic changes in the STA, is rarely reported. Moreover, to the best of our knowledge, none of the previous studies have used IVIM to evaluate the microvascular perfusion status of patients with MMD. Thus, this study intends to evaluate the changes in the cerebral microvascular perfusion status and hemodynamic changes in adult patients with MMD after surgery using 2D PC-MRI and IVIM-MRI without a contrast agent.

## Materials and Methods

This study was approved by the ethics committee of our hospital (No. 2018030). Written informed consent was obtained from each patient before their participation in this study. All experiments were performed in accordance with the relevant guidelines and regulations set by the ethics committee.

### Patients

From December 2017 to January 2019, 33 adult patients with MMD (mean age, 38.61 ± 11.23 years; range, 19–65 years), including 15 women and 18 men, were included in this study. In total, 12 out of 33 patients with MMD had already undergone one side of the combined surgery, and the operation was performed on the other side this time. Detailed clinical information of patients with MMD is shown in Table 1. Preoperative and postoperative blood pressure and blood glucose were carefully monitored for patients with hypertension and diabetes (blood glucose was controlled within the range of 7–9 mmol/L; systolic blood pressure was controlled within the range of 120–130 mmHg). The inclusion criteria were as follows: 1 patients were confirmed to have MMD and were >18 years old; 2 patients with no contraindications in the MR examination; and 3 patients who underwent STA-middle cerebral artery (STA-MCA) bypass and encephalo-duro-myo-synangiosis (EDMS) combined surgery. Herein, we only included patients who underwent combined surgery to avoid the potential bias caused by different surgical types. MMD was diagnosed by DSA according to the criteria of the Research Committee on Spontaneous Occlusion of the Circle of Willis (MMD) of the Ministry of Health and Welfare, Japan (2). The clinical characteristics, including age, sex, and the initial clinical symptoms of the patients, were recorded.

**Table 1:** Clinical information of patients with moyamoya disease (MMD) (33 cases).

	**N**
Age	
Mean age	38.61 ± 11.23 years
Years range	19~65years
Gender	
Male	18
Female	15
Hypertension	12
Diabetes	10
Clinical symptom	
Ischemia	16
Hemorrhage	13
Nonspecific	4
Suzuki stage	
1	0
2	1
3	11
4	12
5	9
6	0
Surgery side	
Left	14
Right	19

### MRI Examination Protocols and Imaging Analysis

All MR examinations were performed with a 3T whole-body MRI scanner (Siemens Skyra Freedom or Siemens Prisma, Siemens Medical Solutions, Erlangen, Germany) using a 32-channel head-neck coil. T1-weighted imaging (T1WI), T2-weighted imaging (T2WI), fluid-attenuated inversion-recovery (FLAIR) sequence, diffusion-weighted imaging (DWI), magnetization-prepared rapid acquisition GRE (T1-MPRAGE), time-to-flight MR angiography (TOF-MRA), PC-MRI, and IVIM imaging were performed for each patient. Detailed MRI scanning parameters are depicted in Table 2. Preoperative MR examinations were performed within 1 week before the surgery, while postoperative MR examinations were performed approximately 2–3 months postsurgery.

**Table 2:** MRI scanning sequences and parameters.

	**TR/TE (ms)**	**Thickness (mm)**	**Flip angle (^**o**^)**	**Intersection gap**	**TA**	**FOV (cm^**2**^)**	**Matrix**	**b-value**
T1WI	230/2.46	5	70	30%	25 s	22 × 22	256 × 192	/
T2WI	5000/117	5	90	30%	54 s	22 × 22	384 × 281	/
FLAIR	8000/85	5	140	20%	1 min 36 s	22 × 22	256 × 162	/
DWI	1300/62	5	192	30%	30 s	24 × 24	192 × 192	0,1000
T1-MPRAGE	2300/2.32	0.9	8	50%	5 min 22 s	26 × 26	256 × 256	/
TOF-MRA	21/3.43	1	18	−18.75%	5 min 13 s	22 × 22	320 × 180	/
2D PC-MRI (pre-scan)	84.6/4.8	6	20	20%	19 s	34 × 34	192 × 68	/
2D PC-MRI	73.08/7.54	4	10	20%	5 min 29 s	18 × 18	336 × 336	/
IVIM	5100/92	5	/	30%	5 min 22 s	22 × 22	130 × 130	16 b values

*TR, time of repetition; TE, time of echo; FOV, field of view; TA, time of acquisition; FLAIR, fluid-attenuated inversion-recovery sequence; DWI, diffusion-weighted imaging; PC-MRI, phase-contrast MRI; IVIM, intravoxel incoherent motion*.

*16 b-value is 0, 20, 40, 80, 110, 140, 170, 200, 300, 400, 500,600, 700, 800, 900, and 1,000 s/mm^2^*.

#### IVIM Scanning Protocol and Imaging Analysis

**Figure 1:**
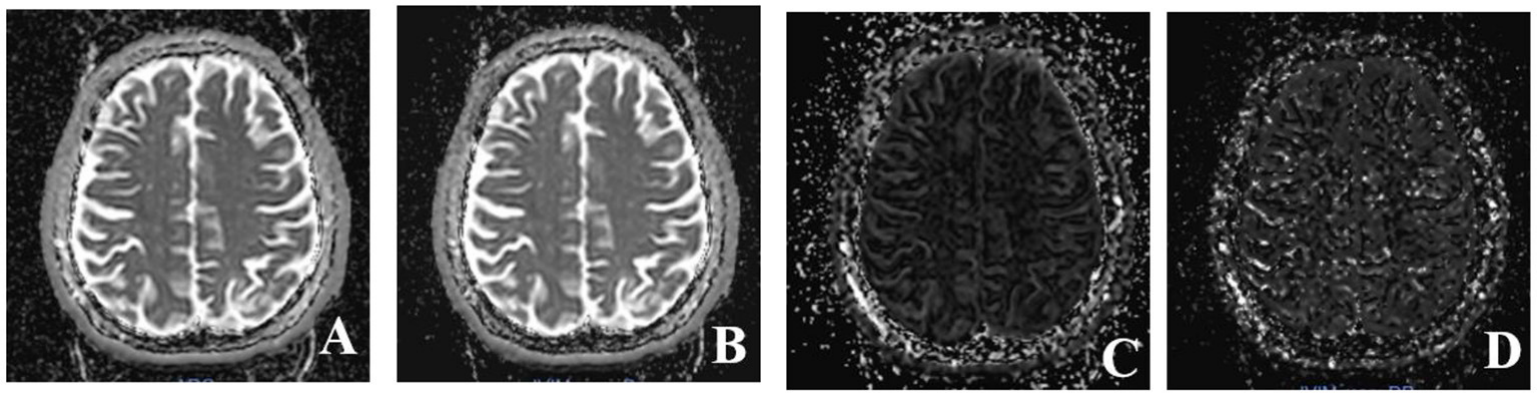
Four parameter maps of intravoxel incoherent motion MRI imaging after post-processing. **(A)** Image of apparent diffusion coefficient (ADC) value. **(B)** Image of *D* value. **(C)** Image of *f* value. **(D)** Image of D^*^ value.

**Figure 2:**
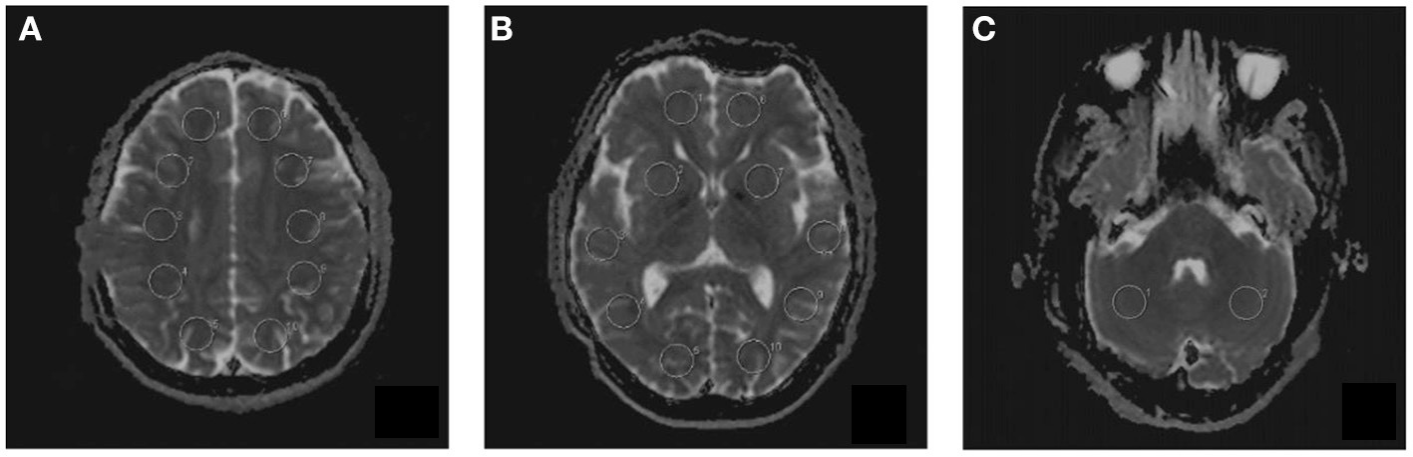
The placement of regions of interest (ROIs) on intravoxel incoherent motion (IVIM) images in three different levels. **(A)** Peripheral white matter level. **(B)** Basal ganglia level. **(C)** Cerebellar hemisphere level. A total of 22 ROIs were placed symmetrically on 3 transversal images.

Multiple *b*-values were used (0, 20, 40, 80, 110, 140, 170, 200, 300, 400, 500, 600, 700, 800, 900, and 1,000 s/mm^2^) in the IVIM sequence. Detailed scanning parameters are listed in Table 2. Four parameters, including the apparent diffusion coefficient (ADC), diffusion coefficient (*D*), pseudodiffusion coefficient (D^*^), and perfusion fraction (*f*), were calculated and derived using a Siemens IVIM work-in-progress package (MR Body Diffusion Toolbox version 1.3.0). The IVIM parameters (*f*, *D*, and D^*^) were calculated by using *Sb*/*S*0 = (1-*f*)·exp (–*b*·*D*) + *f* ·exp (–*b*·D^*^), where *Sb* is the signal intensity of the image at a certain *b*-value, *S*0 is the baseline signal intensity without applying a diffusion gradient. After subtracting the conventional diffusion signal by the algorithm ln, the model can be transformed into a linear model. When *f* and *D* are constants, the small *b*-value interval is linearly fitted by taking ln in a single exponential manner to obtain D^*^, rather than using a double exponential, which is one of the strategies using an algorithm for IVIM (11) (Figure 1). The regions of interest (ROIs) with dimensions of approximately 200 mm^2^ were placed manually, avoiding subarachnoid spaces, cerebral infarction, and cerebral hemorrhage areas. Each cerebral hemisphere was divided into three areas according to the different cerebral supply arteries [anterior cerebral artery supply (ACA) area, MCA supply area, and posterior cerebral artery (PCA) supply area]. A total of 22 ROIs were placed symmetrically on the peripheral white matter, basal ganglia, and cerebellar hemisphere levels (12, 13). To avoid an individual variation, the value of the ipsilateral cerebellar hemisphere was used as a reference to standardize the evaluated parameters. Then, *r*ADC, *rD*, rD^*^, and *rf* were obtained [i.e., rADC = ADC (different blood artery supply area)/ADC (ipsilateral cerebellar hemisphere)] (Figure 2). We selected the ADC map to draw the ROIs, and the placed ROIs were automatically propagated to the other three parametric maps, obtaining *rD*, rD^*^, and *rf*. Each value was measured three times, and the average value was considered as the final result.

#### 2D PC-MRI Scanning Process and Imaging Analysis

**Figure 3:**
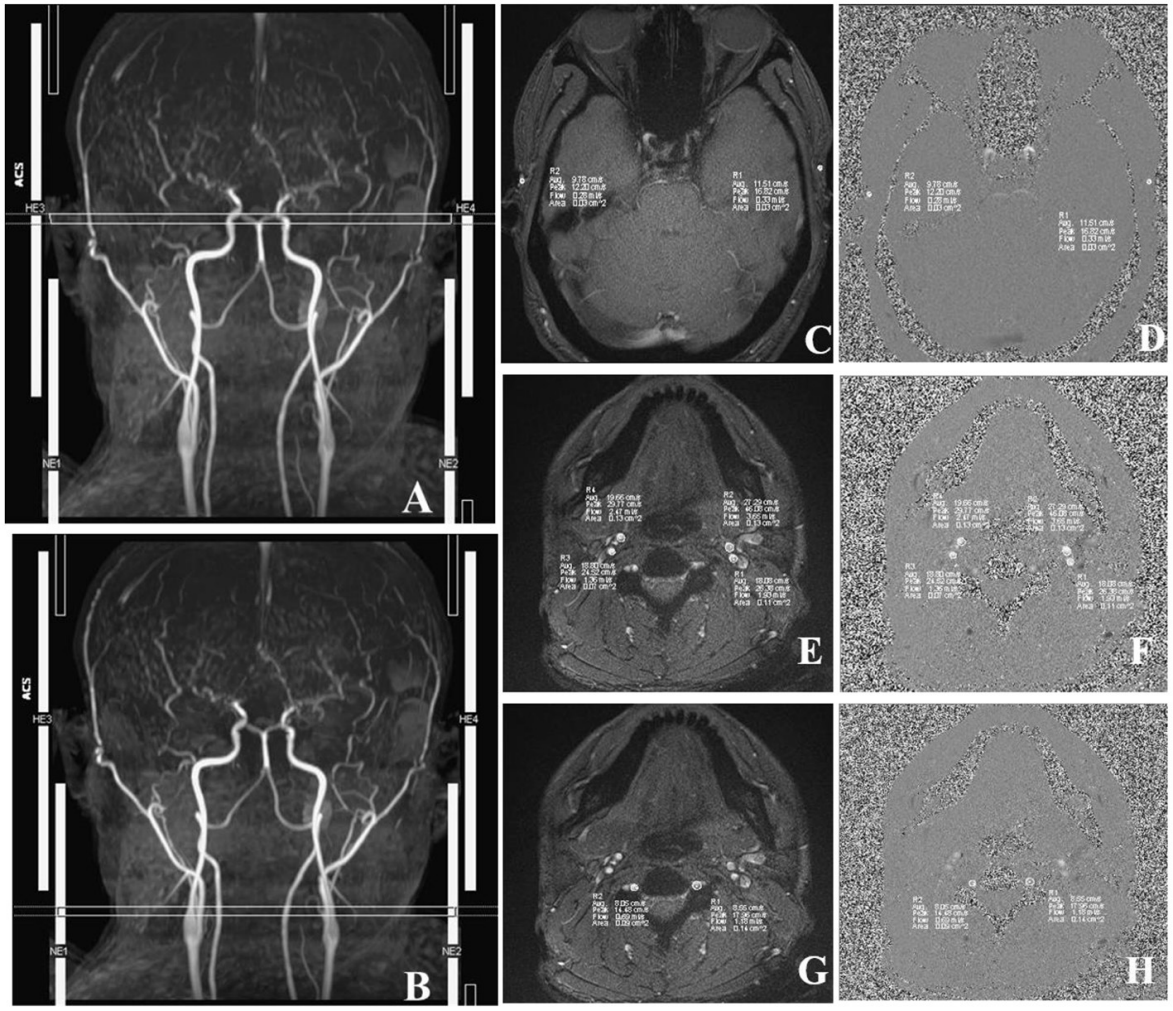
Images acquired in a representative subject illustrate the details of MRI acquisition location and measurement of superficial temporal (STA), internal carotid (ICA), external carotid (ECA), and vertebral (VA) arteries. **(A)** Time-to-flight (TOF) image shows the location and slice position of STA. **(B)** TOF image shows the location and slice position of ICA, ECA, and VA. **(C)** The magnitude image; **(D)** The phase image; *R*1/*R*2, left/right STA. **(E)** The magnitude image; **(F)** The phase image; *R*3/*R*4, right ICA/ECA. **(G)** The phase image; **(H)** The magnitude image, *R*1/*R*2, left/right VA. The circles in *R*1–*R*4 illustrate the voxels with maximal velocities.

First, we used T1-MPRAGE images to locate the 3D-TOF-MRA sequence from the common carotid artery (CCA) level to the top of the skull, and then the PC-MRI sequence was positioned based on the reconstructed MIP image of TOF. In terms of the ICA, external carotid artery (ECA), and VA, the scan plane was placed above the bifurcation of the CCA. In regard to STA, the scan plane was placed in the temporal region, and the cardiac-triggered PC-MRI scans were positioned perpendicular to the target vessels (Figure 3). Second, we performed a prescan to identify the optimal velocity range of target vessels. Specifically, the PC-MRI prescan of the target vessels was performed with the following velocity-encoding strategy: 10, 25, 35, 45, and 60 cm/s for STA and 20, 40, 60, 80, and 120 cm/s for ICA, ECA, and VA. As the optimal velocity range of the targeted vessels for each patient was different, we selected the best velocity range for each patient to perform the final scan based on the results of the prescan (the brightest sequence without any signal lost) to perform the final PC-MRI (Table 2) (14). After finishing the PC-MRI scanning, both magnitude and phase series were loaded into the Argus Viewer (Syngo MR E11). The flow parameters were obtained by drawing ROIs on the magnitude images, which contained the lumen of the vessel as much as possible without exceeding the vessel contour (Figure 3). Finally, the results for velocities, flows, volume, and cross-sectional areas of arteries are reported in the summary table of Argus.

### Grouping and Comparison

**Figure 4:**
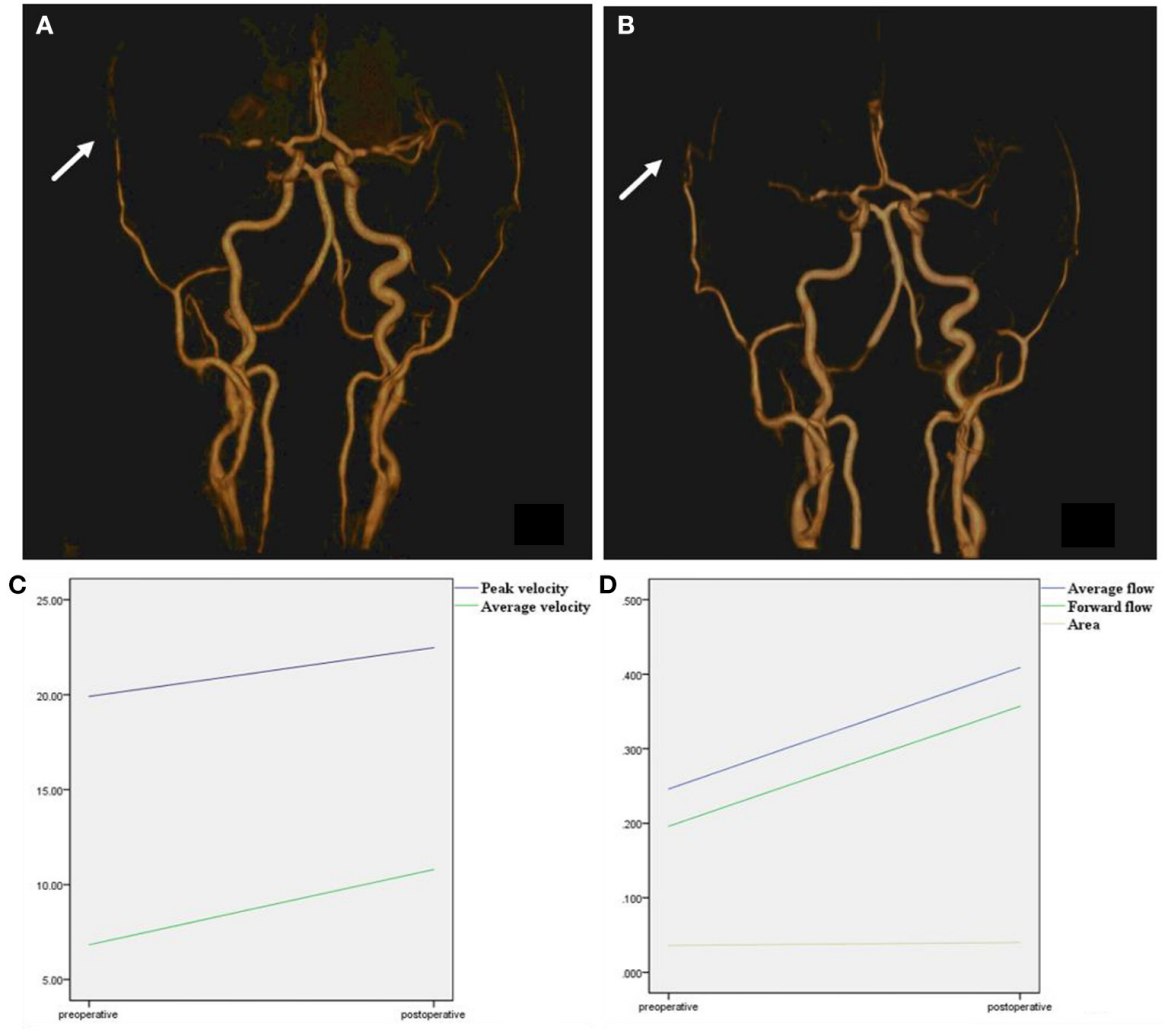
Time-to-flight image before and after surgery. **(A)** TOF image before surgery, right superficial temporal artery was narrow before surgery (arrow). **(B)** TOF image after surgery, after combined surgery, the anastomoses vessel can be seen (arrow). The right superficial temporal artery was thickened compared with a preoperative image. **(C)** Peak velocity and average velocity of the right superficial temporal artery were significantly enlarged after surgery. **(D)** Average flow, forward flow, and area of the right superficial temporal artery were significantly enlarged after surgery.

All preoperative and postoperative parameters in different hemispheres were compared (Figure 4). Additionally, the IVIM parameters were compared between the same cerebral artery supply area in the two hemispheres (ipsilateral/surgical side and contralateral/non-surgical side). Furthermore, to investigate the hemodynamic changes in the different types of MMD, patients were divided into hemorrhage and non-hemorrhage groups. Specifically, patients with a previous history of cerebral hemorrhage or showing the features of a cerebral hemorrhage on preoperative MR examination (obsolete cerebral malacic foci with hemosiderosis) were categorized into the hemorrhage group, whereas patients with a history of cerebral infarction and only presenting intracranial lacunar infarction (without hemosiderosis) on preoperative MR examination were classified as non-hemorrhage group.

### Statistical Analysis

An independent *t-*test or the Mann–Whitney U test was used to assess the differences in continuous variables (peak velocity, average velocity, average flow per second, forward volume, and ROI area of different artery vessels; *r*ADC, *rD*, rD^*^, and *rf* of different cerebral blood supply areas). Statistical results were considered as significant when the values of *p* were < 0.05. Statistical analyses were performed using a commercially available computer software program (SPSS 22.0).

## Results

Six (6/33, 18.20%) patients with MMD developed cerebral hyperperfusion syndrome after combined surgery. One (1/33, 0.03%) patient with MMD developed cerebral infarction after combined surgery. All of these patients improved before discharge from the hospital.

### Changes in Postoperative Blood Flow Status in Patients With MMD

The results of the 2D PC-MRI examination are shown in Table 3. The peak velocities of both the ipsilateral and contralateral STAs and the ipsilateral ECA were significantly increased (*p* < 0.05). The average velocities of both the ipsilateral and contralateral STAs were significantly increased (*p* < 0.05). In terms of the average flow and forward volume, all evaluated vessels presented an increasing trend in both hemispheres (*p* < 0.05). Regarding the areas of different vessels, the areas of the ipsilateral STA, contralateral ICA, bilateral ECA, and ipsilateral VA were also increased significantly after surgery (*p* < 0.05), whereas the area of the contralateral VA was decreased (*p* < 0.05). Other assessed hemodynamic values were not significantly altered postsurgery (*p* > 0.05).

**Table 3:** Postoperative hemodynamic changes of patients with MMD (33 cases).

	**Preoperative**	**Postoperative**		***P***
**Ipsilateral (surgery side)**				
Superficial temporal artery				
Peak velocity (cm/s)	14.352 ± 5.922	22.026 ± 10.997	*t* = −3.529	**0.001**
Average velocity (cm/s)	6.841 ± 3.839	11.207 ± 6.880	*t* = −3.183	**0.003**
Average flow (ml/s)	0.097 ± 0.073	0.297 ± 0.296	*t* = −3.773	**0.001**
Forward flow (ml)	0.081 ± 0.058	0.229 ± 0.212	*t* = −3.847	**0.000**
Area (cm^2^)	0.193 ± 0.322	0.481 ± 0.398	*z* = −3.524	**0.000**
Internal carotid artery				
Peak velocity (cm/s)	24.965 ± 10.362	26.848 ± 10.058	*t* = −0.749	0.457
Average velocity (cm/s)	13.415 ± 7.523	12.011 ± 5.637	*t* = 0.858	0.394
Average flow (ml/s)	0.804 ± 0.764	1.293 ± 0.862	*t* = −2.439	**0.018**
Forward flow (ml)	0.665 ± 0.640	1.075 ± 0.742	*t* = −2.403	**0.019**
Area (cm^2^)	0.095 ± 0.147	0.165 ± 0.174	*z* = −2.649	0.008
External carotid artery				
Peak velocity (cm/s)	39.438 ± 13.938	46.710 ± 13.531	*t* = −2.150	**0.035**
Average velocity (cm/s)	16.934 ± 7.806	18.580 ± 6.886	*t* = −0.892	0.376
Average flow (ml/s)	0.933 ± 0.932	2.046 ± 1.168	*t* = −4.276	**0.000**
Forward flow (ml)	0.748 ± 0.715	1.674 ± 0.960	*t* = −4.448	**0.000**
Area (cm^2^)	0.086 ± 0.148	0.163 ± 0.169	*z* = −3.656	**0.000**
Vertebral artery				
Peak velocity (cm/s)	31.797 ± 13.054	34.140 ± 13.265	*t* = −0.723	0.472
Average velocity (cm/s)	17.129 ± 8.771	15.054 ± 6.293	*t* = 1.104	0.274
Average flow (ml/s)	0.826 ± 0.959	1.287 ± 0.982	*z* = −3.072	**0.002**
Forward flow (ml)	0.688 ± 0.832	1.060 ± 0.844	*z* = −2.943	**0.003**
Area (cm^2^)	0.100 ± 0.201	0.129 ± 0.180	*z* = −2.515	**0.012**
**Contralateral (non-surgery side)**				
Superficial temporal artery				
Peak velocity (cm/s)	14.912 ± 6.727	19.964 ± 6.948	*t* = −3.001	**0.004**
Average velocity (cm/s)	7.155 ± 3.329	9.416 ± 4.751	*t* = −2.240	**0.029**
Average flow (ml/s)	0.135 ± 0.193	0.193 ± 0.140	*z* = −3.591	**0.000**
Forward flow (ml)	0.113 ± 0.164	0.147 ± 0.104	*t* = −3.572	**0.000**
Area (cm^2^)	0.432 ± 0.402	0.247 ± 0.369	*t* = −1.830	0.067
Internal carotid artery				
Peak velocity (cm/s)	28.342 ± 13.064	28.519 ± 11.795	*t* = −0.058	0.954
Average velocity (cm/s)	15.694 ± 9.383	12.689 ± 6.439	*t* = 1.517	0.134
Average flow (ml/s)	0.847 ± 0.792	1.797 ± 1.426	*t* = −3.346	**0.002**
Forward flow (ml)	0.668 ± 0.575	1.469 ± 1.210	*t* = −3.435	**0.001**
Area (cm^2^)	0.097 ± 0.161	0.205 ± 0.190	*z* = −3.367	**0.001**
External carotid artery				
Peak velocity (cm/s)	41.436 ± 14.870	46.850 ± 15.406	*t* = −1.452	0.151
Average velocity (cm/s)	17.413 ± 8.986	16.473 ± 5.813	*t* = 0.505	0.615
Average flow (ml/s)	1.051 ± 0.918	1.808 ± 0.061	*t* = −3.098	**0.003**
Forward flow (ml)	0.847 ± 0.711	1.493 ± 0.929	*t* = −3.173	**0.002**
Area (cm^2^)	0.066 ± 0.062	0.140 ± 0.130	*t* = −2.956	**0.004**
Vertebral artery				
Peak velocity (cm/s)	35.331 ± 11.999	37.139 ± 10.909	*t* = 0.640	0.524
Average velocity (cm/s)	19.040 ± 8.089	19.168 ± 17.753	*t* = −0.038	0.970
Average flow (ml/s)	0.789 ± 0.792	1.309 ± 0.772	*z* = −4.181	**0.000**
Forward flow (ml)	0.637 ± 0.630	1.074 ± 0.660	*t* = −2.753	**0.008**
Area (cm^2^)	0.172 ± 0.296	0.131 ± 0.178	*z* = −2.534	**0.011**

*Bold value indicates P < 0.05*.

### Changes in Postoperative Microvascular Perfusion Status in Patients With MMD

The changes among the four IVIM parameters are shown in Table 4. After surgery, only *rf* in the ipsilateral ACA supply area was increased significantly (*p* < 0.05). Furthermore, *rf* in other areas and *r*ADC, *rD*, and rD^*^ in all areas showed no significant changes (*p* > 0.05).

**Table 4:** Postoperative microvascular perfusion changes of patients with MMD (33 cases).

	**Preoperative**	**Postoperative**		*P*
**Ipsilateral (surgery side)**				
rADC-ACA	1.117 ± 0.121	1.118 ± 0.124	*t* = −0.50	0.960
rADC-MCA	1.196 ± 0.151	1.162 ± 0.130	*t* = 0.974	0.334
rADC-PCA	1.200 ± 1.196	1.186 ± 0.166	*t* = 0.315	0.754
rf-ACA	1.012 ± 0.238	1.168 ± 0.277	*t* = −2.432	**0.018**
rf-MCA	1.189 ± 0.238	1.290 ± 0.275	*t* = −1.597	0.115
rf-PCA	1.123 ± 0.303	1.219 ± 0.293	*t* = −1.314	0.193
rD-ACA	1.131 ± 0.120	1.124 ± 0.120	*t* = 0.233	0.817
rD-MCA	1.211 ± 0.154	1.174 ± 0.132	*t* = 1.051	0.297
rD-PCA	1.211 ± 0.190	1.193 ± 0.166	*t* = 0.398	0.692
rD*-ACA	1.072 ± 0.205	1.060 ± 0.212	*t* = 0.235	0.815
rD*-MCA	1.081 ± 0.162	1.091 ± 0.215	*t* = −0.217	0.829
rD*-PCA	0.963 ± 0.155	0.954 ± 0.162	*t* = 0.245	0.807
**Contralateral (non-surgery side)**				
rADC-ACA	1.156 ± 0.110	1.119 ± 0.124	*t* = 1.280	0.205
rADC-MCA	1.176 ± 0.099	1.181 ± 0.103	*t* = −0.209	0.835
rADC-PCA	1.178 ± 0.115	1.175 ± 0.115	*t* = 0.102	0.919
rf-ACA	1.133 ± 0.252	1.077 ± 0.275	*t* = 0.859	0.394
rf-MCA	1.278 ± 0.270	1.215 ± 0.272	*t* = 0.943	0.349
rf-PCA	1.154 ± 0.236	1.119 ± 0.287	*t* = 0.540	0.591
rD-ACA	1.159 ± 0.100	1.127 ± 0.121	*t* = 1.181	0.242
rD-MCA	1.179 ± 0.092	1.187 ± 0.097	*t* = −0.355	0.724
rD-PCA	1.185 ± 0.111	1.184 ± 0.108	*t* = 0.036	0.971
rD*-ACA	1.042 ± 0.179	1.041 ± 0.137	*t* = 0.043	0.965
rD*-MCA	1.088 ± 0.207	1.049 ± 0.165	*t* = 0.854	0.396
rD*-PCA	0.977 ± 0.229	0.926 ± 0.152	*t* = 1.083	0.284

*ACA, anterior cerebral artery; MCA, middle cerebral artery; PCA, posterior cerebral artery. Bold value indicates P < 0.05*.

### Group Analysis of Cerebral Hemodynamic Changes in Patients With MMD

The cohort consisted of 13 cases in the hemorrhage group (mean age, 39.07 ± 9.28 years; 7 women and 6 men) and 20 cases in the non-hemorrhage group (mean age, 38.30 ± 12.56 years; 7 women and 13 men). In the non-hemorrhage group, only *rf* in the ipsilateral ACA supply area was significantly increased after surgery (*p* < 0.05) (Table 5). No significant changes were detected regarding *rf* in other areas (*p* > 0.05), as well as *r*ADC, *rD*, and rD^*^ in all areas (*p* > 0.05) (Table 5). In the hemorrhage group, none of the IVIM parameters showed significant changes postsurgery (*p* > 0.05) (Table 5).

**Table 5:** Postoperative microvascular perfusion changes of non-hemorrhage group (20 cases) and hemorrhage group (13 cases).

	**Preoperative**	**Postoperative**		***P***
**Non-hemorrhage group (20 cases)**				
Ipsilateral (surgery side)				
rADC-ACA	1.112 ± 0.118	1.105 ± 0.146	*t* = 0.172	0.864
rADC-MCA	1.175 ± 0.144	1.134 ± 0.157	*t* = 0.874	0.388
rADC-PCA	1.169 ± 0.172	1.142 ± 0.178	*t* = 0.487	0.629
rf-ACA	0.989 ± 0.193	1.198 ± 0.304	*t* = −2.589	**0.014**
rf-MCA	1.172 ± 0.174	1.266 ± 0.333	*t* = −1.120	0.272
rf-PCA	1.069 ± 0.225	1.193 ± 0.283	*t* = −1.532	0.134
rD-ACA	1.124 ± 0.120	1.108 ± 0.140	*t* = 0.382	0.705
rD-MCA	1.191 ± 0.152	1.136 ± 0.153	*t* = 1.132	0.265
rD-PCA	1.175 ± 0.163	1.148 ± 0.181	*t* = 0.488	0.628
rD*-ACA	1.096 ± 0.221	1.055 ± 0.214	*t* = 0.587	0.561
rD*-MCA	1.130 ± 0.213	1.069 ± 0.213	*t* = 0.912	0.367
rD*-PCA	0.984 ± 0.172	0.916 ± 0.157	*t* = 1.299	0.202
Contralateral (non-surgery side)				
rADC-ACA	1.160 ± 0.125	1.132 ± 0.135	*t* = 0.666	0.509
rADC-MCA	1.177 ± 0.120	1.182 ± 0.108	*t* = −0.154	0.879
rADC-PCA	1.176 ± 0.139	1.178 ± 0.110	*t* = −0.068	0.946
rf-ACA	1.133 ± 0.256	1.084 ± 0.281	*t* = 0.571	0.571
rf-MCA	1.301 ± 0.278	1.230 ± 0.280	*t* = 0.807	0.425
rf-PCA	1.161 ± 0.249	1.121 ± 0.253	*t* = 0.504	0.617
rD-ACA	1.159 ± 0.116	1.141 ± 0.134	*t* = 0.465	0.645
rD-MCA	1.176 ± 0.113	1.188 ± 0.104	*t* = −0.343	0.734
rD-PCA	1.180 ± 0.137	1.187 ± 0.106	*t* = −0.205	0.839
rD*-ACA	1.000 ± 0.165	1.067 ± 0.152	*t* = −1.335	0.190
rD*-MCA	1.049 ± 0.198	1.076 ± 0.195	*t* = −0.432	0.668
rD*-PCA	0.970 ± 0.223	0.918 ± 0.167	*t* = 0.844	0.404
**Hemorrhage group (13 cases)**				
Ipsilateral (surgery side)				
rADC-ACA	1.134 ± 0.117	1.230 ± 0.096	*t* = 0.098	0.923
rADC-MCA	1.241 ± 0.125	1.194 ± 0.112	*t* = 1.004	0.325
rADC-PCA	1.252 ± 0.211	1.250 ± 0.147	*t* = 0.034	0.973
rf-ACA	1.063 ± 0.288	1.108 ± 0.253	*t* = −0.420	0.679
rf-MCA	1.252 ± 0.296	1.292 ± 0.212	*t* = −0.396	0.696
rf-PCA	1.200 ± 0.378	1.268 ± 0.325	*t* = −0.489	0.629
rD-ACA	1.148 ± 0.114	1.140 ± 0.096	*t* = 0.200	0.843
rD-MCA	1.251 ± 0.116	1.222 ± 0.118	*t* = 0.653	0.520
rD-PCA	1.268 ± 0.208	1.260 ± 0.137	*t* = 0.107	0.915
rD*-ACA	1.054 ± 0.224	1.049 ± 0.171	*t* = 0.067	0.947
rD*-MCA	1.061 ± 0.160	1.069 ± 0.141	*t* = −0.135	0.894
rD*-PCA	0.957 ± 0.166	0.987 ± 0.124	*t* = −0.516	0.611
Contralateral (non-surgery side)				
rADC-ACA	1.138 ± 0.082	1.108 ± 0.120	*t* = 0.805	0.429
rADC-MCA	1.168 ± 0.072	1.186 ± 0.089	*t* = −0.572	0.573
rADC-PCA	1.176 ± 0.073	1.177 ± 0.125	*t* = −0.036	0.972
rf-ACA	1.104 ± 0.257	1.093 ± 0.281	*t* = 0.101	0.921
rf-MCA	1.171 ± 0.246	1.265 ± 0.280	*t* = −0.902	0.376
rf-PCA	1.114 ± 0.268	1.144 ± 0.310	*t* = −0.262	0.796
rD-ACA	1.150 ± 0.073	1.114 ± 0.102	*t* = 1.014	0.321
rD-MCA	1.177 ± 0.061	1.191 ± 0.081	*t* = −0.500	0.622
rD-PCA	1.188 ± 0.058	1.183 ± 0.115	*t* = 0.131	0.897
rD*-ACA	1.056 ± 0.170	1.053 ± 0.152	*t* = 0.053	0.958
rD*-MCA	1.064 ± 0.187	1.089 ± 0.175	*t* = −0.354	0.726
rD*-PCA	0.972 ± 0.247	0.953 ± 0.136	*t* = 0.244	0.810

*ACA, anterior cerebral artery; MCA, middle cerebral artery; PCA, posterior cerebral artery. Bold value indicates P < 0.05*.

## Discussion

In this study, we evaluated the postoperative hemodynamic changes of adult patients with MMD by using a PC-MRI and IVIM sequence. Our results showed that PC-MRI is a potential modality to accurately and non-invasively assess the postoperative changes in the blood flow status in the bilateral STA, ICA, ECA, and VA. The microvascular perfusion status could be evaluated by IVIM, and *rf* in the ACA supply area was significantly increased in the non-hemorrhage group.

Superficial temporal is one of the target vessels of vascular reconstruction surgery, indicating the importance of assessing the hemodynamic changes that directly reflect the treatment effect of STA-MCA anastomosis. Previous studies using duplex ultrasonography showed that the mean blood flow velocity and diameter of the operated STA increased after extracranial–intracranial bypass surgery compared to the baseline values (15, 16). The results of our study were consistent with previous studies. Specifically, our results showed that the peak velocity, average velocity, average flow, forward volume, and area of the ipsilateral STA were increased after surgery (*p* < 0.05), indirectly suggesting that STA-MCA anastomosis could increase the intracranial blood supply by the ECA system. STA is one of the terminal branches of ECA and has substantial circulatory resistance. The natural circulatory resistance of the distal STA could be reduced after bypass surgery (15), resulting in an increased lumen, velocity, and volume of the vessel. Moreover, after bypass surgery, the blood supply of the ipsilateral cerebral hemisphere depends on STA to a large extent. This circumstance might be a driving factor to elevate the velocity and increase the flow of the ipsilateral STA. The STA is also one of the terminal branches of the ECA. Therefore, it is understandable that the hemodynamic changes in the STA may partially reflect the flow status of the ECA. As expected, the peak velocity, average flow, and forward volume of the ipsilateral ECA were all significantly increased after surgery (*p* < 0.05).

Except for the blood flow changes of the ipsilateral STA and ECA, the average flow and forward volume of the ipsilateral ICA and the average flow, forward volume, and ROI area of the contralateral ICA, ECA, and VA were also significantly increased (*p* < 0.05). This could be explained by the complex blood supply status in patients with MMD. Hemodynamic statuses of the bilateral hemispheres compensate and affect each other through Willis circles and collateral vessels, resulting in a comprehensive blood flow status in bilateral hemispheres. A recent study showed that the mean transient time might be shortened within 1 week after surgery (17). Over a period of time after surgery, the cerebral blood supply might be gradually adjusted, and the total blood supply of the brain would increase.

An accurate evaluation of the changes in the microvascular perfusion status of patients with MMD after surgery is clinically important. IVIM can assess the microperfusion status, which is based on the fact that blood microperfusion causes signal attenuation at low *b*-values (18). A previous study used IVIM as a potential modality to evaluate microvascular perfusion, especially using the parameter *f* (10, 19). Federau et al. (20) used IVIM to investigate the hemodynamic changes in patients with acute infarction, and the results showed that the perfusion fraction *f* was significantly decreased in the infarction area. In this study, only *rf* of the ACA supply area in the ipsilateral hemisphere was significantly increased after surgery (*p* < 0.05). The STA-MCA bypass combined with EDMS was performed for all the patients in our study, and *the rf* of the MCA supply area was supposed to improve significantly. The blood flow from STA-MCA bypasses may drain into the ACA supply area *via* collateral vessels. However, an increasing trend for *rf* in MCA and PCA supply areas was observed in the ipsilateral hemisphere. The perfusion fraction *f* reflects the signal fraction of capillary blood flow in the entire water molecule diffusion pool within each voxel (21). After surgery, the formation of small collateral vessels of the ipsilateral hemisphere increased, resulting in an increase in *f*. As it is shown earlier, several flow indicators of the contralateral ICA, ECA, STA, and VA were increased after surgery; however, *rf* of the contralateral hemisphere showed no significant changes. This might indicate that the improvement of microperfusion status may not be present in 2–3 months or that the initial microperfusion status may be less affected than blood flow before surgery. Cerebrovascular reactivity (CVR) was decreased in patients with MMD, and unilateral revascularization improved the CVR of the ipsilateral hemisphere. However, whether surgery can affect the contralateral hemisphere, CVR is controversial. Further deterioration (22), almost no effect (23), and a slight improvement (24) have been reported. Therefore, the perfusion status of the contralateral hemisphere is hard to evaluate due to the controversial points of CVR.

In terms of *r*ADC, *rD*, and rD^*^, no significant changes were detected in either ipsilateral or contralateral hemispheres after surgery (*p* < 0.05), which might be decided by their different functions. The ADC and *D* values reflect the diffusivity of water in biological tissues. The pseudo different coefficient D^*^ represents both water movement in the blood flow and diffusion motion within a single voxel, which may have a potential value in the evaluation of microvascular perfusion status (19, 25, 26). However, the results are controversial (10, 27, 28). A previous study showed that the repeatability of the D^*^ value was poor (27). This may be because the D^*^ value is highly sensitive to capillary flow and volume effects in any region with cerebrospinal fluid (CSF) filling or necrotic space, and D^*^ maps had a lower signal-to-noise ratio than the other maps (29).

Furthermore, to investigate whether there were differences in the microvascular changes in the subtype of patients with MMD, an IVIM analysis was performed in two separate groups: non-hemorrhage and hemorrhage. In this study, 39.39% (13/33) of patients were categorized into the hemorrhage group, which was in accordance with a previous study and accounted for 21–56% of MMD (30). The current study showed that an improvement in microvascular perfusion was more obvious in the non-hemorrhage group than in the hemorrhage group. Revascularization surgery is recommended for patients with ischemic MMD, with the aim of ameliorating microvascular perfusion (29). In patients with hemorrhagic MMD, the main purpose of surgery is to reduce the circulation pressure of collateral vessels and then to prevent recurrent intracranial hemorrhage rather than to increase the blood supply of the brain (29). The current study also showed that microvascular perfusion was significantly improved in the non-hemorrhage group but not in the hemorrhage group. This phenomenon might be explained by abundant collateral vasculature, which also indicated that preoperative microvascular perfusion status was better in patients with hemorrhagic MMD than in patients with ischemic MMD. Therefore, the incremental microvascular perfusion after surgery in patients with hemorrhagic MMD is not as obvious as in patients with ischemic MMD.

Nevertheless, the present study has several limitations. First, as the sample size was small, additional data are required to verify our results. Another drawback is the lack of comparison with other imaging methods for validation, such as TCD, BOLD-CVR, ASL, and DSC-PWI. However, the two modalities were widely used in other clinical scenarios, indirectly verifying the robustness of our methods (31, 32). Nonetheless, a validation analysis with other conventional methods will be performed in our future study. Third, our study only evaluated postoperative MR examinations after surgery, while the longitudinal changes in the cerebral blood supply were not analyzed and need to be improved in a future study.

## Conclusion

The current study showed that 2D PC-MRI and the parameter *rf* derived from IVIM imaging could potentially and non-invasively evaluate the changes in hemodynamic status in patients with MMD after surgery. Moreover, an improvement in the microvascular perfusion status is more obvious in patients with ischemic MMD than in patients with hemorrhagic MMD.

## Data Availability Statement

The raw data supporting the conclusions of this article will be made available by the authors, without undue reservation.

## Ethics Statement

The study was approved by the Ethics Committee of Huadong Hospital (No. 2018030). The patients/participants provided their written informed consent to participate in this study.

## Author Contributions

WZ, FG, and GL contributed to the conception and design of the study. WZ, FG, YZ, SL, and JL organized the database. WZ and FG performed the statistical analysis and wrote the first draft of this manuscript. YZ, SL, YD, and ZZ wrote the sections of this manuscript. All authors contributed to manuscript revision, read, and approved the submitted version.

## Funding

This study was funded by the National Natural Science Foundation Project (Grant No. 81771816), National Natural Science Foundation of China (Grant No. 82102157), Hunan Provincial Natural Science Foundation of China (Grant No. 2021JJ40895), the Science and Technology Innovation Program of Hunan Province (Grant No. 2020SK53423), and the Clinical Research Center for Medical Imaging in Hunan Province (Grant No. 2020SK4001).

## Conflict of Interest

The authors declare that the research was conducted in the absence of any commercial or financial relationships that could be construed as a potential conflict of interest.

## Publisher's Note

All claims expressed in this article are solely those of the authors and do not necessarily represent those of their affiliated organizations, or those of the publisher, the editors and the reviewers. Any product that may be evaluated in this article, or claim that may be made by its manufacturer, is not guaranteed or endorsed by the publisher.
